# ‘The Devil’s Company’: A Grounded Theory Study on Aging, Loneliness and Social Change Among ‘Older Adult Children’ in Rural Indonesia

**DOI:** 10.3389/fsoc.2021.659285

**Published:** 2021-06-22

**Authors:** Julia Schröders, Mark Nichter, Miguel San Sebastian, Maria Nilsson, Fatwa Sari Tetra Dewi

**Affiliations:** ^1^ Department of Epidemiology and Global Health, Umeå University, Umeå, Sweden; ^2^ School of Anthropology, College of Social and Behavioral Sciences, The University of Arizona, Tucson, AZ, United States; ^3^ Department of Health Behavior, Environment and Social Medicine, Faculty of Medicine, Public Health and Nursing, Gadjah Mada University, Yogyakarta, Indonesia

**Keywords:** loneliness, social networks, elderly care, rural aging population, social change, intergenerational relations within a family, grounded theory analysis, Indonesia

## Abstract

**Introduction:** As a consequence of rising life expectancies, many families are no longer made up of one, but two simultaneously aging generations. This elderly parent–older adult child (OAC) dyad has emerged as a newly overserved yet little explored demographic phenomenon. Studies on this intergenerational aging dyad and the possible ramifications of when caregivers are simultaneously aging with care-receivers are scarce, especially in low and middle-income countries. This study explored the process by which rural Indonesian OACs experience their own aging, thereby gaining insights into how this newly evolving reality impacts the traditional ways of old-age care provision.

**Methods:** This study has a qualitative design and draws on eight focus group discussions with 48 community-dwelling OACs (23 men, 25 women; mean age 64 years) in four rural villages in the Yogyakarta Special Region, Indonesia. The theoretical framework was largely inspired by symbolic interactionism aided by the sensitizing concepts of social network deficits, interpersonal emotions, and the social construction of risks. Data were analyzed using Grounded Theory as outlined by Corbin and Strauss.

**Results:** Respondents’ accounts reflected four categories: 1) aging in a welt of chronic insecurity; 2) OACs: a generation “betwixt and between” expected demands and unmet expectations; 3) landscapes of loneliness; and 4) compromising against conventions. As depicted in a conceptual model, these categories interrelated with each other and were linked by a core category, “bargaining for a sense of security”, which collectively summarized a process by which OACs’ experienced their own course of aging.

**Conclusion:** Our study provided insights into how and why loneliness emerged amidst the challenges of social and demographic transformations and how in response to this unconventional compromises were made, which affect both the networks of caretakers and the places of old-age care. It is doing so by including the perspectives of rural Indonesian OACs. The results showed how multiple intersecting negative experiences constrained the aging experiences of OACs and produced precarious aging trajectories. Our findings highlight the importance of old-age loneliness as an emerging public health and social problem by discussing how intrinsically this emotion was interwoven with social life.

## Introduction

Social networks are important social determinants of health influencing various health outcomes across the life course (Berkman et al., 2000; Ertel et al., 2009; Holt-Lunstad et al., 2015; Kelly et al., 2017; Schröders et al., 2020), as well as an important resource when it comes to navigating health care systems (Valtorta et al., 2018; Schröders et al., 2021). Particularly in low and middle-income countries (LMICs) with limited social welfare protection, social networks appear to be even more influential, oftentimes ensuring the only chances for health and wellbeing for many vulnerable populations (Kelly et al., 2014; Perkins et al., 2015). Especially for older people, social networks have become important resources for coping with late-life adversities and enhance social and economic resilience against a range of adverse events. In the absence of coordinated health and social welfare policies, regulated state-run pension schemes, formal geriatric services or long-term care (LTC) models, various forms of informal - mostly family-based - social networks stand out as the backbone for old age care provision - an insurance which is resting globally on diverse cultural, religious or customary commands, values, and norms (United Nations Population Division, 2005). However, in recent years, many countries have been undergoing two ‘megatrends’ which have critically impacted the provision of these ensuring and insuring informal safety nets.

The first comprises of population aging combined with health transitions, i.e., transformations of disease occurrence and cause of death towards chronic non-communicable diseases (NCDs) as well as a substantial increase of the disability burden from NCDs (He et al., 2016; World Health Organization, 2018). Globally, population aging has led to increased life expectancies (LE) and age-structure alterations. Particularly in LMICs, both LEs at birth and at older ages have continuously improved over the past decades and demographic projections suggest that by 2050 the number of persons aged 65 and over will more than double so that LMICs will host more than two-thirds of the world’s older populations. By 2050, Eastern and South-Eastern Asia will be hosting the largest share (37%) of older people while also showing the highest gender gap in LE at age 65 (3.4 years). Coupled with population aging is the rapidly growing burden of NCDs, which is particularly visible in LMICs where NCDs have become the leading cause of death (59.7%) and disability (50% of all disability-adjusted life years; DALYs) (Murray et al., 2020; Vos et al., 2020; World Health Organization, 2018). The largest amount (85%) of NCD deaths occurs prematurely between the ages of 30–69 years. Data shows that the risk of prematurely dying from a NCD is highest in LMICs (47% of all NCD deaths), particularly in the South-East Asian region (50% of all NCD deaths) (Adioetomo and Mujahid, 2014; Abbafati et al., 2020; Murray et al., 2020; Vos et al., 2020).

As a consequence of longevity and premature mortality, today, many families are facing the burdens of two simultaneously aging generations, instead of one (Vaupel et al., 1998; Herm et al., 2012; Jopp et al., 2016). Coined in the literature as a “newly-observed wrinkle in the demographic of aging families” (Kim et al., 2018), studies are beginning to focus on simultaneously aging elderly parents with “children” who are themselves in their sixties and already facing varied aging-related health and social needs. Despite its increasing global occurrence, this new phenomenon, the older adult child (OAC)—elderly parent dyad, has so far received relatively little scientific attention apart from studies in the United States (Poon et al., 1992; Randall et al., 2010; Kim et al., 2018) and Germany (Boerner et al., 2016). However, to the best of our knowledge, no study in a LMIC setting has focused on this intergenerational aging dyad and the possible multifaceted ramifications of when caregivers are simultaneously aging with care-receivers.

Rapid social change constitutes the second “megatrend”. Globally, decreasing fertility rates and the rise of childlessness has resulted in a smaller network of caretakers being available for aging populations. Additionally, and particularly in LMICs, rising trends of rural-to-urban and international labor migration, as well as the increased involvement of women in the labor market, have substantially drained care-taking resources from aging parents’ care networks, particularly in rural areas. Many studies have also reported on changing normative views on filial caretaking obligations and intergenerational reciprocity, particularly in Asia (Kreager and Schröders-Butterfill, 2004; Mehta and Ko, 2004; Hashimoto et al., 2005; Canda, 2013). This challenges the persistent view that younger generations, i.e., mostly one’s own children, are in any case willing and able to handle the various old-age care needs of their elderly parents. All these social changes are either creating *de facto* or *de jure* holes in older people’s social networks and may combine or exacerbate in various ways and effect and affect the prevalence of social network adversities among aging populations.

We further argue that the interconnectivity and interplay of these two “megatrends” will not only have devastating effects on older adults’ individual physical, mental and social wellbeing but will also have far-reaching meso and macro-level consequences for existing family structures and intergenerational ties, and hugely impact the demands for effective policies and programs for older people’s health and welfare, and chiefly for the provision of formal LTC and other geriatric services. Particularly LMICs—where close and extended kin networks have worked (and are still largely working) as essential “safety nets” in old age, are poised to facing the realities of rising chronic health problems among aging populations amidst concomitant rapid social change. In such contexts, the emergence of the OAC-elderly parent dyad will create a disproportionally growing need to ensure the wellbeing and welfare of two simultaneously aging generations with similar, yet distinct, health and social needs.

Transitioning from purely informal family-based old-age care arrangements to state-run sustainable and equitable LTC and geriatric care models will be a major challenge for health systems in most LMICs. There is a well-developed evidence base from high-income countries on the transition from informal to formal care arrangements, i.e., on the balancing of informal and formal care services or the degree of state participation (Litwin and Auslander, 1992; Sundstrom et al., 2002; Lowenstein and Daatland, 2006; Sundström et al., 2006; Litwin and Attias-Donfut, 2009; Motel-Klingebiel et al., 2009). But despite containing over 60% of the global 65 + population, only 5% of research studies originate from LMICs reflecting that aging-related health and welfare policies and LTC models remain a neglected issue in both the policy and research agendas in many LMICs (Lloyd-Sherlock, 2014). Despite repeated calls to align health systems with the Global Strategy Plan on Ageing and Health and to adequately meet the needs of aging populations, many countries fail to do so amidst efforts to provide Universal Health Coverage (UHC) and progressing towards achieving the Sustainable Development Goals (SDGs) (World Health Organization, 2017).

In South-East Asia, Indonesia currently stands at a critical juncture facing the double burden of these two “megatrends”. The country is undergoing distinct demographic and epidemiological transitions (Arifin and Ananta, 2013; Bappenas, 2013; Adioetomo and Mujahid, 2014; Arifin et al., 2016; Schröders et al., 2017), all in a context of rapidly alternating health and aging policies (Pisani et al., 2017; Agustina et al., 2019). Globally, Indonesia has the fifth-largest elderly population. The share of people aged 60 + years will more than double from 2019 to 2050 rising from 10 to 21%; particularly with increasing LEs, older women will make up almost 12% of the total population by 2050, rising from 5% in 2019 (Bappenas, 2013; Adioetomo and Mujahid, 2014; Arifin et al., 2016). The country is also experiencing an “aging of the older population” as LEs at age 60 have been likewise rising over the past decades steadily increasing the share of the oldest old (80 + years) population (Arifin and Ananta, 2013; Adioetomo and Mujahid, 2014). Population aging challenges are not only mirrored in these age-structural changes which are foreshadowing the emergence of a growing number of OAC-elderly parent dyads, but also in various dependency measures reflecting the increasingly complicated and seemingly unsustainable and inequitable interplay between older and younger generations. Old-age-dependency ratios are projected to increase from 7.6 to 15.6 during the coming years to 2035 (Adioetomo and Mujahid, 2014; Agustina et al., 2019). In the absence of public old-age financing schemes, older Indonesians–similar to many parts in South and South-Eastern Asia–rely highly on assets and labor income to finance old-age consumption. Labor force participation past retirement age (i.e., 57 years in 2016) remains high. More than 40% of all 70–79-year-olds and nearly 23% of all 80+-year-olds continue working (Adioetomo and Mujahid, 2014; Pisani et al., 2017). Nonetheless, old-age poverty remains a challenge, disproportionally affecting women and older adults in rural areas (Ananta, 2012; Priebe and Howell, 2014; Bloom et al., 2015). An epidemiological transition towards NCDs and disability is likewise progressing swiftly in the country with an increase in NCDs from 52% in 2000 to 75% in 2018 (World Health Organization, 2018; Schröders et al., 2017; Institute for Health Metrics and Evaluation (IHME), 2018; The National Institute of Health Research and Development and Indonesia Ministry of Health, 2018). Though Indonesia launched an UHC scheme in 2014, many challenges for the provision for formal LTC and geriatric services remain, i.e., lack of LTC regulations, institutionalized programs, and integration of services, particularly in underserved rural areas (Mboi, 2015; Pisani et al., 2017; Agustina et al., 2019). In the context of relatively weak formal networks of protection, our previous work has highlighted the importance of informal social networks for affecting both older adults’ health (Schröders et al., 2020) and their healthcare utilization (Schröders et al., 2021).

When considered disjointedly, a robust evidence base exists on issues addressing epidemiological transitions and population aging or aspects of social change and elderly care in LMICs. However, to the best of our knowledge, currently there is no information available from studies addressing these issues in any way conjointly and much less with a focus on the possible ramifications stemming from two simultaneously aging generations. Particularly in rural LMIC settings (where, as a general rule, formal networks are weak but informal networks appear strong), a holistic understanding of how “megatrends” like population aging and social change converge and shape older adults’ perceptions and experiences of aging will however be essential for comprehending the challenges not only for individuals’ health and wellbeing, but also for family structures and functions, and the ability of communities and governments to provide adequate resources for support. In addressing this paucity, the aim of this study was to obtain an initial, yet nuanced, understanding of these challenges through the perspectives of OACs residing in rural Indonesian settings. Ultimately, the findings from this study can help generating more detailed future research questions and serve as a basis for designing effective interventions and policy responses.

## Materials and Methods

### Overall Study Design and Conceptual Framework

This study is part of a larger mixed-method research project investigating the role of social networks for older adults’ health in the transitional setting of Indonesia. Following a convergent parallel design, in the larger project, both quantitative and qualitative data were collected from community-dwelling older adults in select rural and urban areas of the Yogyakarta Special Region during autumn 2016. Further details about the project have been reported elsewhere (Schröders, 2021). Here, we present results of the rural sub-study, conducted in the Gunung Kidul regency.

The present qualitative study used Grounded Theory for several reasons. First, because of its usefulness for understanding specific phenomena (e.g., loneliness) within dynamic processes (e.g., aging amidst social changes) using eclectic data (Langley, 1999). Second, because Grounded Theory describes and conceptualizes respondents’ views, actions and life experiences within the context in which they live, it ensures a participant-centered understanding giving voice to the community (Tweed and Charmaz, 2012). Third, Grounded Theory is particularly useful when theory and research are under-developed and underdefined (Strauss and Corbin, 1998). Because little is known about OAC’s in general and their aging process in particular, Grounded Theory appeared to be the most appropriate qualitative approach to guide the analysis of this study.

Our Grounded Theory study was initially underpinned by a Goffmanian perspective of symbolic interactionism which is resting on the assumptions that humans do not simply react to events but respond based on their understanding and interpretation of those events and that society is preserved and created through repeated interactions between individuals (Goffman, 1978; Blumer, 1986). This perspective was particularly chosen for its ability to link agency (i.e., the individual agent exercising intentional action) and structure (i.e., social contexts that define the range of potential actions) through social interaction (Goffman, 1978) as we aimed to describe the process by which OACs experience their own aging in the context of social change.

As a methodological consequence of the previous, we used a systemic perspective on social networks as a sensitizing concept for exploring possible lines of inquiry (Strauss and Corbin, 1998; Charmaz, 2014). Such a perspective led us to the various ecological levels (i.e., individual, intra- and intergenerational family, community, society) of OACs’ networks. Besides this systemic perspective, we also employed a narrower conceptualization of social networks which we subsumed under the umbrella term “social network deficits”. This involved being sensitive to structural deficiencies (e.g., network losses, shrinking, holes or isolation), functional inadequacies (e.g., support failures, perceived loneliness), and shortfalls in social network quality (e.g., negative exchanges, conflicts, strains or social exclusion). The focus on functional and quality aspects was aided by being sensitive to respondents’ expressed feelings and emotions. For this, we leaned towards approaches from the “sociology of emotions” area as well as emerging attempts to theorize “emotional capital”, which again aided bridging the agency-structure divide by linking individual resources and processes to macro-structural forces (Thoits, 1989; Bericat, 2016). Later, during the process of following the procedures of constant comparison (Glaser and Holton, 2004; Holton, 2010) for our Grounded Theory approach, particularly the fourth level comparing our emerging theory with existing literature, we additionally utilized perspectives on emotions’ centrality to macro-social processes, i.e. ranging from Durkheim’s thesis on the social construction of emotions (Fisher and Chon, 1989), Keltner’s and Van Keef’s work on the social functions and effects of emotions (Keltner and Haidt, 1999; Van Kleef, 2016), and aspects of Ahmed’s “cultural politics of emotions” (Ahmed, 2014).

While symbolic interactionism-informed foci on social networks, particularly network deficits and the emergence of loneliness as a central interpersonal emotion, aided the emergence of several categories, still during later stages of coding and analyzing the data, it appeared necessary to engage with social constructivism as an additional perspective. Particularly the “social construction of risk” appeared central in developing the core category and saturating aspects of other (sub-)categories (Arnoldi, 2009; Burgess et al., 2014). Social constructivism can be seen as an extension of symbolic interactionist theory and proposes that reality is cognitively constructed and that social constructs develop based on social interactions (Berger et al., 1991). Social constructivism brings in a stronger focus on power relations and meanings and has therefore oftentimes been adopted to understand deviance, i.e., by juxtaposing the construction of commonly accepted norms against the labelling of behaviors or emotions as inappropriate, sometimes by attaching stigma (Luhmann, 2005; Kleinman and Hall-Clifford, 2009; Adler and Adler, 2011). By applying such a perspective, we were able to theorize and delineate how in our data, network deficits, which emerged through the (non)interaction with social influences, appeared as socially constructed risks and this has further enabled us to theorize loneliness as a stigmatizing emotion. Subsequently, the social constructivist focus on power relations enabled the development of the core category, which essentially embodied a process of “bargaining for a sense of security” and as such encompassed both aspects of power relations and risk construction. Many have emphasized on the natural linkage between a social constructivist perspective and a “social problem” perspective (Blumer, 1971; Kitsuse and Spector, 1987). A “social problem” perspective perceives a social problem not as a static condition, but as an activity, and by this sets analytical focus less on the actual causes of a social problem but on the processes by which a certain group or society defines specific conditions as social problems (Kitsuse and Spector, 1987). In our study, such analytical focus let to the unfolding of a process by which OACs experienced their own aging voiced in a Grounded Theory of “bargaining for a sense of security”.

### Sampling and Study Setting: Rural Gunung Kidul

**FIGURE 1:**
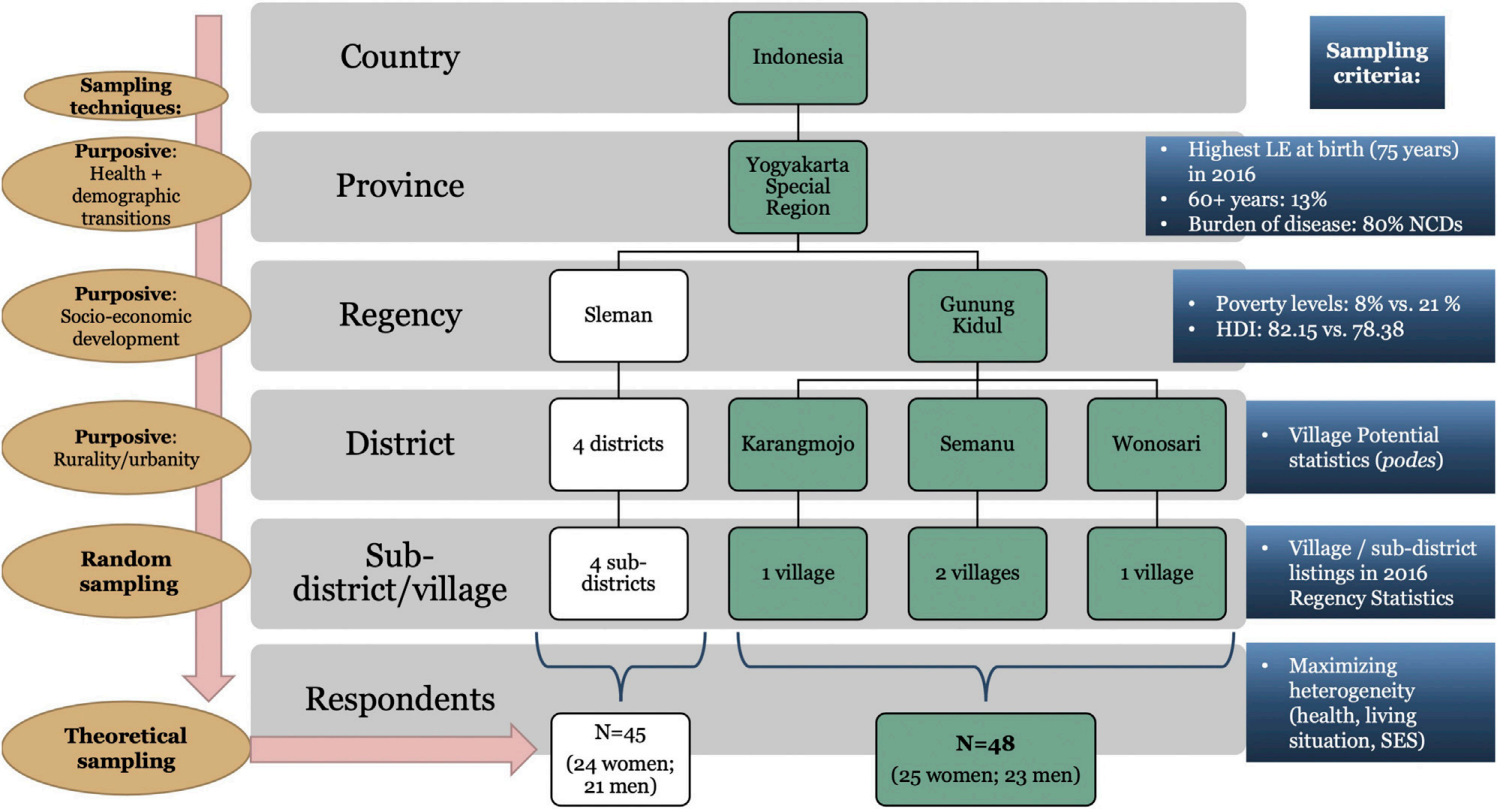
Overview of sampling strategy (Schröders, 2021).

The study areas were drawn using a combination of non-probability and random sampling techniques (Figure 1). Purposive sampling of the Yogyakarta Special Region was based on inter-provincial differences in LEs, age-structure compositions, and the burden of NCDs (Schröders, 2021).

For selecting study areas below regional level, we used socio-economic development together with the concepts of rurality/urbanity. Based on the notion that in LMICs in general (Perkins et al., 2015) and in Indonesia in particular (Schröders et al., 2020) the presence or absence of social networks make a pronounced difference to the ultimate consequences of poverty on people’s lives, we decided to purposively sample two regencies which vary significantly with regards to their poverty levels and human development indices (HDI): the Sleman regency ranking best within the region with a poverty rate of 8.21% and a HDI of 82.15; and the Gunung Kidul regency ranking last with 19.34% of its population being classified as poor and a HDI of 67.82 [Badan Pusat Statistik (BPS) D. I. Yogyakarta (Statistic Office of the Yogyakarta Special Region), 2018]. Many studies have shown that poverty in Indonesia is strongly related to the socio-cultural tendency of poor people to share their limited assets—a practice that makes people poorer as their social networks expand (Geertz, 1963; Alexander and Alexander, 1982).

In the next stage, considering the spatial dimensions of poverty and following research stating that rural and urban social networks are structurally and functionally distinctive and differentially influence how people cope with economic and other circumstances (Klärner and Knabe, 2019), we purposively selected three rural districts within the Gunung Kidul regency and four urban districts within the Sleman regency. For this, we followed the official urban-rural classification based on Village Potential (*podes* ^1^) statistics [Badan Pusat Statistik (BPS) Indonesia (Statistics Indonesia), 2016].

**FIGURE 2:**
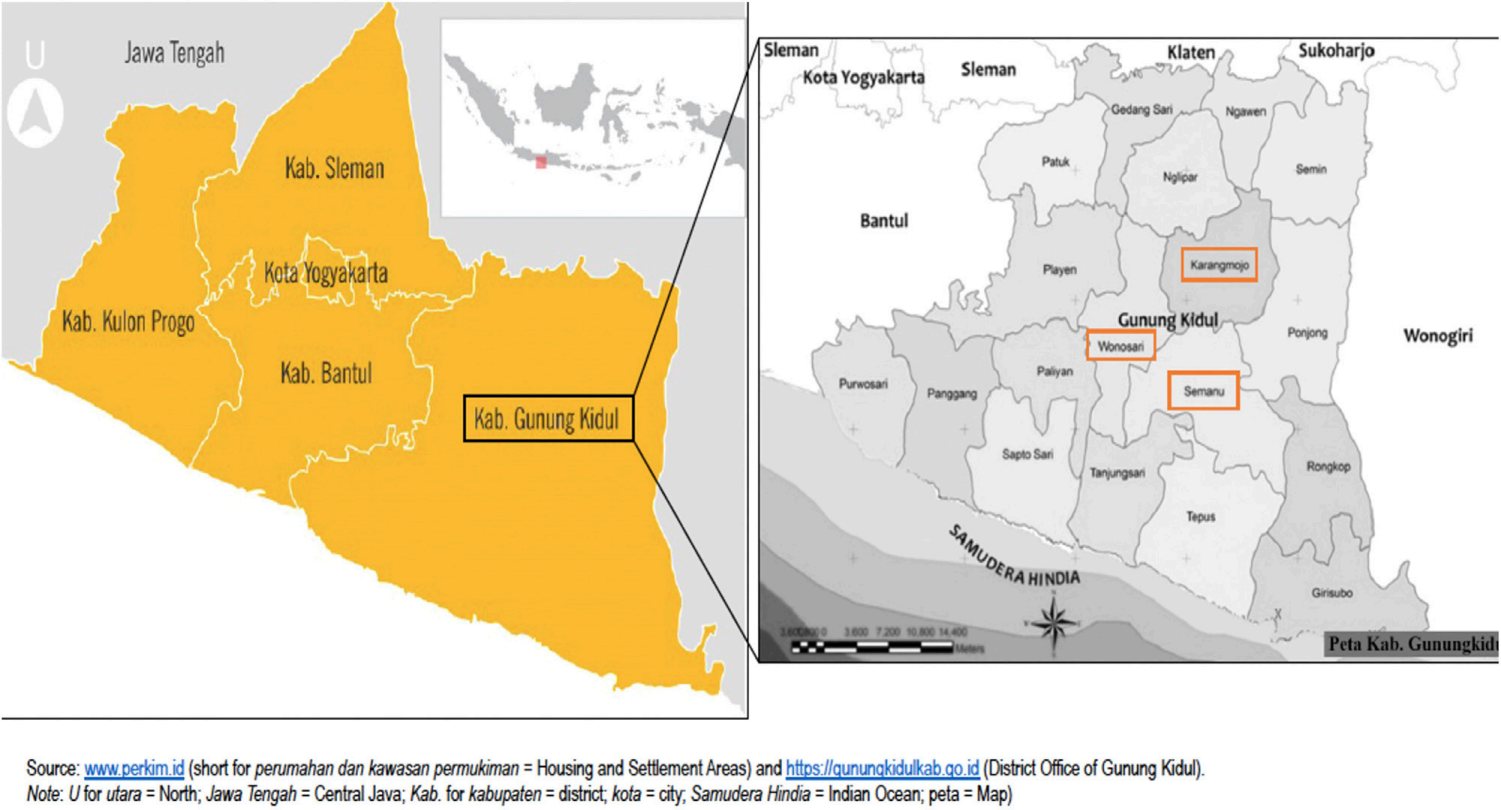
Research locations: four rural villages in the Semanu, Karangmojo and Wonosari districts in the Gunung Kidul Regency, Yogyakarta Special Region, Indonesia.

As a last sampling stage, villages in each district were randomly selected from the 2016 Regency Statistics [Badan Pusat Statistik (BPS) Kabupaten Gunungkidul (Badan Pusat Statistik (BPS) Kabupaten Sleman (Statistic Office of the Sleman Regency), 2016; Badan Pusat Statistik (BPS) Kabupaten Sleman (Statistic Office of the Sleman Regency), 2016]. The present study includes four villages in rural Gunung Kidul: one village each in the Wonosari and Karangmojo districts and two villages in the Semanu district. For confidentiality reasons, Figure 2 shows the research locations only down to district level.

### Recruitment of Participants

Our recruitment strategy involved approaching the districts’ community health care centers (*puskesmas*) and invite users of the *posyandu lansia* program (integrated service center for the elderly). After approaching the districts’ health offices, we subsequently got in touch with the villages’ community health workers, so-called *kaders*. We orally and in writing informed the district health offices and the *kaders* about the aim of our study, how data will be collected, and explained measures to ensure participants’ anonymity, and the confidential handling of data. Following that, each *kader* advertised our study in their monthly *posyandu lansia* program and recruited each 6–8 OACs above the age of 50 years with varying levels of health issues (i.e., ranging from reporting no health issues to patients struggling with multiple chronic morbidities) and living situations (i.e., ranging from respondents co-residing with several family members to widows/widowers living alone) for our study. Being a predominantly rural regency, which is facing continuous water shortages, famines, poor infrastructure, and sluggish economic growth, it was not possible to sample respondents with very contrasting socioeconomic profiles. The respondents, the *kaders* and the research team together scheduled a suitable time and place for conducting the focus group discussions (FGDs). In Gunung Kidul, the described sampling and recruitment strategy rendered 48 respondents for eight FGD sessions. Preparatory field work and data collection took place between August and October 2016.

### Data Collection

All data were collected by the first author (JS) and two fieldwork assistants under local supervision of the last author (FSTD). Due to the language barriers of the first author, a modified interpreter-assisted FGD approach was chosen (Barbour, 2008; Quintanilha et al., 2015). Involving a real-time interpreter who translates the content of the FGDs as they occur to the researcher was regarded as the best means to allow the first author to take an active role in the data generation process. The FGDs were moderated by a native Indonesian and Javanese speaking fieldwork assistant who had several years of experience in qualitative data collection in the health and development work field. A second fieldwork assistant, a local medical student, who likewise is a native Indonesian and Javanese speaker with a good proficiency in English acted as the real-time interpreter for the first author. Both fieldwork assistants and the first author were women.

In total, we conducted eight FGDs in rural Gunung Kidul. The sessions were held separately for men and women. This decision was based on our previous experiences of conducting research in this setting where a homogeneous group composition encouraged respondents to talk more freely and openly particularly when the topics were sensitive, as well as on our ambition to capture gender-specific views. For six FGDs we borrowed the local village community halls; two FGDs were conducted in participants’ private homes. FGDs lasted between 42 and 85 min (mean 63 min) and were audiotaped and later transcribed verbatim.

We utilized a semi-structured topic guide which was designed based on Harrell and Bradley’s “funnel approach” starting with *grand tour* questions allowing respondents to share autobiographical accounts of themselves and their social context (Harrell and Bradley, 2009). Subsequently, questions were funneled into more focused inquiries on respondents’ embeddedness in existing kin and non-kin social networks; their health status and how it interposes with their social networks and social activities; their views on the family’s vs state’s role and responsibility for old age care and their views and experiences with loneliness.

The field work was arranged in a way that allowed for data collection and preliminary analyses to occur simultaneously (Corbin and Strauss, 2014). To capture a wide variation of experiences and to build in extra heterogeneity into our sample, we purposefully sampled participants to reflect a wide range of health conditions, living arrangements and to some degree their socioeconomic background. Peer-debriefings after each FGD led to alterations and additions to the topic guide and emergent preliminary findings and interpretations guided theoretical sampling choices during the fieldwork period.

Data collection continued until theoretical saturation was reached—which was being defined as the point where no new conceptual insights with regards to our research question were emerging (Speziale et al., 2011; Charmaz, 2014). We reached this stage after the seventh FGD but to ensure no new categories would occur, we conducted one additional FGDs with a mixed group containing both men and women. Field notes and memos were written by the first author throughout data collection and analysis stages. More analytical, theoretical, and conceptual memos were kept and reviewed throughout the coding stage and the writing of the manuscript (Montgomery and Bailey, 2007).

Since most participants were of Javanese ethnic background the language used during the FGDs was heterogeneous, containing bilingual Indonesian and Javanese talk. All FGDs were audio recorded, transcribed verbatim and translated into English by the FGD moderator, who is an Indonesian and Javanese native speaker with high English language proficiency. The de-identified English transcripts were checked for accuracy and then uploaded into NVivo (version 12 for Mac) for further analysis.

### Data Analysis and Theory Development

Initially during the field work, we tried to follow an iterative process of preliminary analysis, sampling and data collection occurring concurrently and informing each other. Later, the analysis of the eight FGD transcripts was carried out following the principles of Grounded Theory as outlined by the latest edition of Corbin and Strauss (Corbin and Strauss, 2014). The coding procedure involved consecutive sessions of open, axial, and selective coding.

Open coding involved a detailed examination of the transcribed texts and decomposing them line-by-line to uncover emerging concepts. By drawing constant comparisons, conceptually similar codes were grouped into categories. In the first round of open coding an effort was made to make limited abstractions and to stay close to the text assigning predominantly *in-vivo* codes reflecting respondents’ (translated) voices. As categories became more saturated, properties were defined and located along a dimensional continuum.

Axial coding represents the process of re-constituting the data by using a conceptual paradigm model. The paradigm model is an analytical model used to link categories and sub-categories in a set of relationships at a higher level of abstraction. The coding paradigm—similar to Glaser’s “six C’s” coding family (Glaser and Strauss, 1967)—focused on the following aspects of the phenomenon under study: causal condition(s); context(s); action(s), interaction(s) (including emotions of people in response to the phenomenon); intervening condition(s); and the consequence(s) (Strauss and Corbin, 1998; Corbin and Strauss, 2014).

The aim of the final selective coding [later also referred to as “theoretical integration” (Corbin and Strauss, 2014)], was to further integrate categories to create and refine a conceptual model grounded in the data. This was done by identifying a core category which should represent the main concern of the study, interact significantly with the other categories and was sophisticated enough to account for the complexity and nuances within the data. Eventually, the results of this study are expressed as a substantive theory articulated in a grounded conceptual model.

**FIGURE 3:**
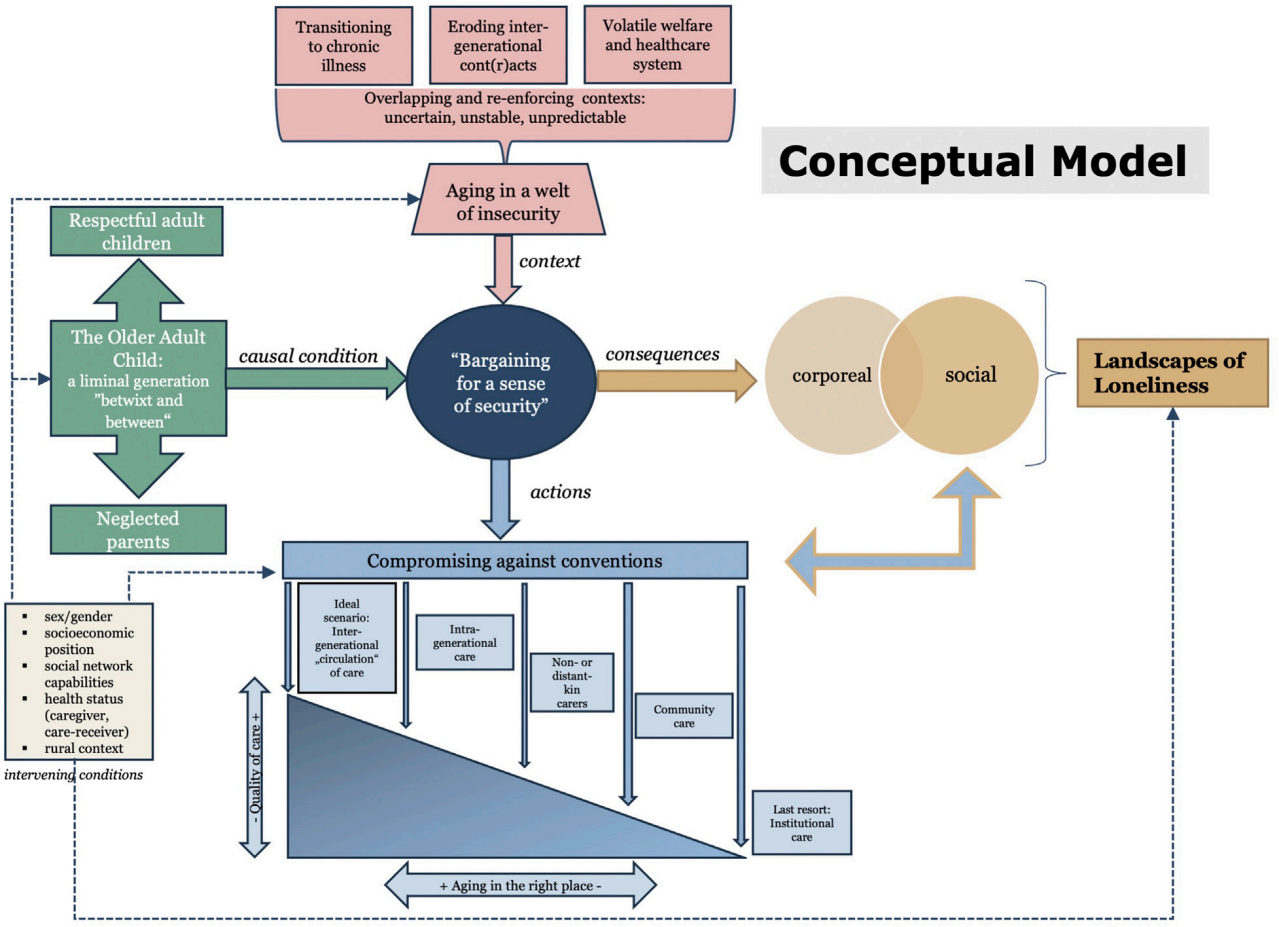
‘Bargaining for a sense of security’—a conceptual model how ‘older adult children’ experience their aging.

Using diagrams (the final one shown in Figure 3) and reviewing memos were two tools that aided the emergence of the core category. By focusing on the core category and by trimming off excess and filling less developed categories until they were saturated, a clearly defined storyline reflected in the conceptual model was created. Another tool that aided the articulation of the storyline was the use of a “conditional (or consequential) matrix”, which is an analytic device to stimulate thinking about micro, meso, and macro conditions affecting the main phenomena (Corbin and Strauss, 2014). In the present study, this was deemed as especially helpful as the matrix pertains to the multi-dimensional and multi-contextual nature of social networks. This matrix particularly enabled saturation of the categories and sub-categories describing the “context” and “consequences”.

Following Glaser and Holton’s suggestion, four levels of constant comparison were applied, i.e., comparing codes with codes, codes with emerging categories, categories with one another, and the emerging theory with the literature (Glaser and Holton, 2004; Holton, 2010).

### Rigor and Trustworthiness

We adopted Lincoln and Guba’s strategies to increase this study’s trustworthiness for credibility, dependability, confirmability and transferability (Lincoln and Guba, 1985). Table 1 summarizes the applied strategies for each trustworthiness criterion. In order to assist readers to evaluate the rigor and comprehensiveness of our Grounded Theory study, we report according to the COREQ checklist (COnsolidated criteria for REporting Qualitative research (Tong et al., 2007).

**TABLE 1:** Trustworthiness strategies and procedures.

Criterion	Main strategies	Procedures applied to achieve rigor
Credibility	Prolonged and varied engagement with study setting	-All researchers had previous experience with health research in Indonesia
		-First author (JS) had continued engagement on site to get acquainted with setting
		-Native Javanese researcher (FSTD) and field work assistants to ensure cultural sensitivity during data collection and linguistic authenticity during data interpretation
		-Involvement of community health workers and district health offices
	Establishing researchers’ authority	-All researchers had previous track record of health research in Indonesia, particularly central Java
		-Multidisciplinary research team (e.g. anthropology, epidemiology, health promotion, indigenous health, medicine, psychology, public health) ensuring eclectic theoretical knowledge and investigative skills
	Regular peer debriefings	-Regular triangulation sessions during data collection and analysis process
		-Regular debriefings with field work assistants
	Credible data collection process	-FGD guide was pilot tested
		-Semi-structured procedure and funneled approach to interviewing allowed for focus and flexibility
		-Interpreter assisted FGD approach ensured active involvement of first author during data collection
		-Native Indonesian and Javanese speaking FGD moderator who was skilled in qualitative field work and data collection
		-Sex/gender stratified FGDs
Dependability	Dense description of study methods	-Detailed description of data collection and analysis process, including any emergent designs and cyclic processes of data collection and analysis
		-Use of quotations to exemplify how findings were grounded in data
	Data authenticity	-Review of verbatim and translated transcripts against audio files to ensure accuracy
		-Cultural and linguistic clarifications provided by transcriber
		-When necessary, back-translation of key quotations
		-Ensuring a balanced use between interpretations and supporting quotations; avoiding overrepresentation of quotes from single FGDs
Confirmability	Reflexivity	-Researcher reflexivity
		-First author kept reflective memos during data collection and analysis
		-Reflections on sensitive issues and potential biases
	Triangulation	-Methodological and data source triangulation for the larger project (i.e. the mixed method approach)
		-Four levels of constant comparisons and theoretical triangulation during data analysis
Transferability	Sampling techniques	-Combination of different sampling techniques (non-probability, random, theoretical sampling) to ensure heterogeneous group of participants with varying level of experiences
	Data saturation	-Data saturation/informational redundancy reached after conducting 7 FGDs
		-Operational saturation (i.e. decreasing number of emerging codes over time) and theoretical saturation (saturation of categories and sub-categories) reached during data coding and analysis
	Description of study setting and participants	-Details on demographic and epidemiological profile of study area and health and socio-economic situation of participants

### Ethical Considerations

Prior to study start, prospective participants were informed about the study’s objectives, verbally and in writing. Participation was voluntary and participants were informed about their right to withdraw from the study at any time without stating reason(s). FGDs were conducted and audio taped after participants gave written informed consent to participate and for the collection of personal data. For the two illiterate participants, oral consent was documented by a witness (a geriatric nurse and the village head). All invited participants provided written informed consent and were reimbursed 50.000 IDR (ca. 4 USD) for their time. None of the participants withdrew their participation.

To guarantee anonymity and minimize as best as possible the chances of subsequent deductive disclosure, all potentially identifying personal information was removed, i.e., participants’ and third-party names, as well as the respective villages they reside in. Data was analyzed at group (i.e., FGD) level. Accordingly, quotations were attributed not to pseudonymized participants but to non-stigmatizing categories of participants and indicating the FGD session. Participants were ensured about means to assure external confidentiality, that is confidential data usage by the researchers. Vice versa, and in the interest of preserving internal confidentiality, participants were urged to keep the content of the FGDs for themselves.

The study was conducted in accordance with the Declaration of Helsinki. Ethical approval for the study was obtained at the Medical and Health Research Ethics Committee (MHREC), Faculty of Medicine, Gadjah Mada University; and Dr. Sardjito General Hospital, Yogyakarta, Indonesia (Ref: KE/FK/096/EC/2016). Research permits were issued by the Regional Government of the Yogyakarta Special Region and the municipalities of Sleman and Gunung Kidul, in which the project was conducted.

## Results

Results are based on eight FGDs with 48 OACs residing in four rural villages in the Gunung Kidul regency in the Yogyakarta Special Region, Indonesia (Figure 2). In total, 25 women and 23 men, who were on average 64 years old, participated in the FGDs. On average, there were six participants per FGD. Eleven respondents were widowed. Many respondents—like most of the villagers—were engaged in agricultural-related occupations. We included both land-owning smallholding farmers and socioeconomically less-advantaged agricultural laborers, who in many cases had to rely on additional non-agricultural forms of income to finance their rural livelihoods. Most women were housewives with limited formal occupational engagement. Some women supplemented household incomes with one or more income-generating activity, i.e., with trading agricultural goods or handicraft production. Many respondents continued working beyond the official retirement age (i.e., 57 years in 2016) due to the lack of formal pension or social protection schemes. Most respondents (56%) did not achieve education beyond primary school. On average, respondents had 3.3 children and lived in households with 3.9 persons. Women reported more chronic conditions and rated their health worse compared to men. Further details are presented in Table 2 which provides an overview of respondents’ characteristics.

**TABLE 2:** Respondents’ characteristics.

	Total (48, 100%)	Women (25; 53%)	Men (23; 47%)
Age, mean in years	64	63	65
50–59	16 (33)	12 (48)	4 (17)
60–69	22 (46)	8 (32)	14 (61)
70–79	7 (15)	3 (12)	4 (17)
80+	3 (6)	2 (8)	1 (5)
Marital status			
Married	37 (77)	18 (72)	19 (83)
Widowed	11 (23)	7 (28)	4 (17)
Occupation			
Casual worker in agriculture	19 (40)	8 (32)	11 (48)
Farmer, land owning	10 (21)	5 (20)	5 (22)
Self-employed	8 (17)	7 (28)	1 (4)
Retired	7 (15)	1 (4)	6 (26)
Homemaker	3 (5)	3 (12)	n/a
Government employee	1 (2)	1 (4)	n/a
Education			
None	8 (17)	6 (24)	2 (9)
Less than primary school	3 (6)	n/a	3 (13)
Primary school	16 (33)	9 (36)	7 (30)
Secondary school	10 (21)	6 (24)	4 (18)
High school	8 (17)	2 (8)	6 (26)
College, university	3 (6)	2 (8)	1 (4)
Number of children [mean (SD)]	3.3 (1.4)		
Range	1–7		
Household size [mean (SD)]^a^	3.9 (1.7)		
Range	1–8		
Self-rated health			
Good	15 (31)	7 (28)	8 (35)
Moderate	19 (40)	9 (36)	10 (44)
Bad	14 (29)	9 (36)	5 (21)
Chronic health condition^b^			
Yes	17 (35)	10 (40)	7 (30)
No	31 (65)	15 (60)	16 (70)

a Two respondents lived alone (household size = 1); ten respondents (21%) lived together with their partners (household size = 2).

b Includes self-reported physician diagnoses of arthritis and rheumatism; asthma, high blood glucose and diabetes; cardiovascular conditions including hypertension, strokes; high cholesterol; hyperuricemia and chronic kidney disease; vertigo. Six respondents reported multiple chronic conditions.

During the stages of analyzing the data for concepts, 822 open codes emerged from the transcripts and subsequently formed seven categories and 26 sub-categories. Most codes were relating to elderly care, intergenerational relations, socio-cultural change, loneliness, and age-related changes in respondents’ health. Analyses were subsequently developed around the core category “bargaining for a sense of security” and resulted in the emergence of four categories which are linked by and interact with the core category. The four categories are as follows: 1) “This era has passed”: aging in a welt of insecurity (*context*); 2) “Older adult children”: a generation “betwixt and between” expected demands and unmet expectations (*cause*); 3) “The devil’s company”: landscapes of loneliness (*consequences*), and 4) “For sure, there will be gossip”: compromising against conventions (*actions*). Although the occurrence of these experiences and processes and the order in which they appear seem to apply quite homogeneously among our respondents, we also noticed large individual differences based on the influence of a number of intervening conditions. Any reference to intervening conditions describes how vulnerabilities in terms of respondents’ sex/gender, socioeconomic position, social network capabilities, health status, and the rural context influence, i.e., enable or disable, their abilities and possibilities to “bargain for a sense of security”.

The core phenomenon, the four categories and their sub-categories are presented in Table 3. From these findings, a conceptual model (Figure 3) depicting the associations and interactions of the four categories with each other and their relations to the core category was developed. The following sections describe each of the four categories and provide a synoptic narrative of the conceptual model and the substantive Grounded Theory of OACs’ aging as a process of “bargaining for a sense of security”. Results are based on the top-to-bottom order in the model from *context* to *actions* via *cause* and *consequences*. Quotes from respondents were integrated into the narrative of our findings to exemplify and substantiate how our results were grounded in the data.

**TABLE 3:** Overview of the core category, categories, and sub-categories and their position within the paradigm model.

Sub-categories	Categories		Core category
• “It limits us in many ways”: Transitioning to uncertain states of chronic illness and disability	“This era has passed”: Aging in a welt of insecurity	Context	Bargaining for a sense of security
• “We are left behind”: Sociocultural change eroding stable intergenerational cont(r)acts			
• “Asking for assistance is difficult”: Navigating a volatile and unpredictable welfare and healthcare system			
• “It is our duty”: Remaining a respectful child	Older adult children: A generation ‘betwixt and between’ expected demands and unmet expectations	Cause		
• “Let’s just hope for the best”: Fearing to become a neglected parent				
• “There is no medicine for such a pain”: Loneliness as corporeal suffering	“The devil’s company”: Landscapes of loneliness	Consequences		
• “I was not a human anymore”: Loneliness as a social stigma			
• “But is doesn’t feel comfortable”: Compromising on quality of care	“For sure, there will be gossip”: Compromising against conventions	Actions		
• From “home care” to “care home”: Spatial compromises in securing care				

### “This Era Has Passed”: Aging in a Welt of Insecurity (*Context*)

This first category is describing the contextual circumstances in which the core phenomenon and the other categories are embedded in. Overall, respondents’ accounts can be synthesized in the form of three overlapping and reinforcing contextual factors that together map a “welt of insecurity” in which respondents are growing old in: 1) individual (micro-) level transitions towards uncertain states of chronic illness and disability; 2) sociocultural change eroding previously stable meso-level intergenerational relationships for old age care provision; and 3) on the macro-level, a volatile and unpredictable welfare system.

In our data, we found various accounts of how the context of growing old in today’s “*sophisticated times*” was clearly delimited from the past “*good old times*” they themselves grew up in and saw their own parents (and oftentimes grandparents) grow old in. From these accounts, it became evident that aging was mostly viewed as a negative experience by our respondents and characterized by frequently re-occurring and reinforcing unsatisfying and unanticipated turns in many areas of their life. It also appears that aspects of “chronification” related to all levels within their “insecure welt”, i.e., the data shows how transient conditions translate into persistent states of insecurity. Therefore, both the feelings of aging in chronic insecurity together with the temporal juxtaposition of past vs. present and old vs. young appeared to be central in unfolding the *context* of this study. In this sense, notions of nostalgia, which was a common way of our respondents to express their discontent with many of todays’ conditions and their longing for an “*(…) era (that) has passed*” (Man from Wonosari, FGD 4) was also a linguistic indicator for such adverse processes of “chronification”.

#### “It Limits Us in Many Ways”: Transitioning Towards Uncertain States of Chronic Illness and Disability

Health, or the absence of it, was described as a major source of uncertainty in respondents’ lives. Frequently comparing their generations’ health with their same-aged parents (and grandparents), they noted that “*in the past they didn*’*t even know the kinds of diseases that we have now*” (Woman from Karangmojo, FGD 7). Almost all respondents reported suffering from a NCD. Asthma, diabetes, hypertension and stroke were the most commonly reported diseases among themselves and within their villages. Many respondents suffered from multiple NCDs concurrently and functional impairments and disabilities were a major source of health-related uncertainty.

This uncertainty was commonly experienced and described as a rollercoaster ride, “*there are ups and downs*” (Man from Semanu, FGD 2) and “*good days and bad days*” (Woman from Karangmojo, FGD 7). Many expressed a chronic sense of unease and worry as they often times could not predict how their health would be the next day. This particularly applied to those suffering from silent diseases which had no obvious “*warning (signs)*” (Woman from Wonosari, FGD 3), strong symptoms, or did not convey an active picture of sickness and suffering that could be observed by others. This finding particularly unfolded when comparing respondents with different diseases (e.g., those with arthritis vs. those with hypertension) and their narratives on how (un)successfully they could negotiate and enquire help from their families, in particular their children.

Particularly arthritis, rheumatism, vertigo, and sensory impairments such as vision problems and hearing loss created a lot of concern, worry, and uncertainty since they limited respondents’ ability to perform their everyday tasks and activities. Those performing physical labor or working in agriculture to make a living were deeply worried about the limiting effect of their poor physical health on their ability to work and earn a living. Many would also deliberately reduce their social contacts and limit their social activities feeling uncomfortable and ashamed of their diseases or disabilities. For example, one woman was apprehensive of her asthmatic breathing as the “*breath and sound will disturb the other people around*” (Woman from Semanu, FGD 1); another male respondent with limited hearing ability felt too ashamed to continue joining prayers or community gatherings where many people would talk to him.

NCDs were experienced as incapacitating and disabling, thus, in our data, medications were frequently discussed as means to alleviate symptoms, pain and also dependency. However, for most respondents taking medication for one or several diseases was not a straightforward undertaking. A major challenge was that due to the chronic nature of the disease, medication needed to be taken regularly, as one respondent observed: “*I must keep consuming it. It ( = the disease) comes back every time I run out of medicine*” (Woman from Wonosari, FGD 3). Further, the main obstacle to regular medication was the fact that they were highly depended on a support network, particularly their children, to be able to access and utilize healthcare services and purchase necessary drugs.

Health-related uncertainty and constantly re-occurring dependency on medication and their children put respondents into uncertain physical and economic conditions which can potentially deteriorate their present situation. Many respondents expressed a feeling of “*being limited in many ways*” (Man from Wonosari, FGD 4) by their deteriorating health and this is being reinforced by eroding support networks (sub-category *“We Are Left Behind”: Sociocultural Change Eroding Stable Intergenerational Cont(r)acts*) and a difficult-to-navigate healthcare system (sub-category *“Asking for Assistance Is Difficult”: Navigating a Volatile and Unpredictable Welfare and Healthcare System*).

#### “We Are Left Behind”: Sociocultural Change Eroding Stable Intergenerational Cont(r)acts

The most frequently discussed source of instability was pertaining to sociocultural changes, specifically expressed by the gradual erosion of previously stable intergenerational contacts and contracts between the respondents and their children. Our respondents hold deeply embedded cultural expectations for intergenerational care provision which are imbedded in various Javanese traditions, Confucian norms, and religious (here predominantly Sunni Islam) commands. The intergenerational contract is based on the logic of debt, where parents raise their children as debtors as one respondent exemplified: “*It*’*s like paying back the breastmilk*” (Man from Wonosari, FGD 4). Making clear comparisons between past and present, respondents gave several examples reifying this eroding process.

The decline in nuclear households and children’s outmigration from rural areas to the cities for employment was a major concern of many older parents, as one respondent noted, “*I seldom meet my children even though none of them is married yet. Both have a very busy schedule (…) They are far away. They live in the city in their own houses.*” (Woman from Semanu, FGD 1). Even though (due to increased life expectancies) more generations are living at the same time compared to the past, physical and geographical distance between family members has increased. Connected to this, respondents expressed strong concerns about a widening gap between the generations due to the acquisition of new attitudes, values and behaviors among the younger generation. Respondents’—representing the older generations’ viewpoint—noted that “*(…) there are many (children) who respect their parents, but also many who do not anymore*” (Man from Semanu, FGD 6). Many mentioned instances of the “*anak nakal*”, the “disrespectful child” and highlighted that “*young people in the past weren*’*t like that*” (Man from Karangmojo, FGD 8). Respondents clearly expressed that many among the younger generation were breaking the previous generational contract even though “*it was agreed upon*” (Man from Wonosari, FGD 4).

While many examples referred to eroding filial obligations in the sense of instrumental or emotional support from children to their older parents, many respondents expressed also feelings of economic instability. For example, redirecting financial resources away from the older generation towards grandchildren, a third generation involved in this generational interplay, was described as a common new practice disrupting the old contract: “*they (= respondents*’ *children) need a lot of money for the schools, for the tuition fees. So, I should not expect much.*” (Woman from Wonosari, FGD 3). However, only few respondents would admit that their children had been acting “*disrespectful*” towards them, i.e., breaking with the traditional norms and practices of filial piety is nothing to be spoken publicly about. Most instances referred to by hearsay to “*a person in another village*”. Particularly in times of a health crisis or economic difficulty (and oftentimes these go hand in hand) not receiving support from ones’ own children is being reflected upon as an embarrassing and shameful situation. Some respondents would explain the erosion of the contract as individual failures—abandoning the idea that this was inevitably caused by ongoing sociocultural change and a side-effect of modernity explaining that “*it all depends on how you educate your children*” (Woman from Semanu, FGD 5), while others reflected upon this from a broader generational perspective. Many respondents thought that their own generation was “*old-fashioned*” and “*outdated*” and that it was difficult for them to maintain a meaningful connection with the younger generation even though they tried. Especially older women struggled with this intergenerational dialogue and keeping up with younger women’s increasing familiarity and involvement with Western and modern culture and values: “*The young people talk about top models, cellphones and other things we don*’*t understand. We like to talk about the price of shallots in the market or what we should cook for dinner. Kitchen things…old stuff. So, we are quiet and just sit and listen to them*.” (Woman from Wonosari, FGD 3).

Our data reified a detailed picture of how sociocultural change has negatively impacted traditional Javanese family structures, the frequency and quality of intergenerational contact between our respondents and their children (and grandchildren) and subsequently led to the erosion of the previously stable and reliable intergenerational contract in this rural Indonesian setting. Unstable arrangements with regards to their own old-age care provision was communicated through nostalgic and exclusionary language making clear demarcations between past and present and young and old. Many respondents expressed that their children and grandchildren were unreliable and unstable sources of support and “*don*’*t have enough time*” for their parents because of today’s “*stressful life*” (Woman from Wonosari, FGD 3). In this sense, respondents clearly recognized structural, functional and quality-related network deficits as socially produced risks in their everyday lives. Due to normative ruptures, diminishing filial piety and an increasing cleavage in interest between generations, many respondents felt being pushed towards the periphery of their family networks “*feeling left behind*” (Woman from Karangmojo, FGD 7)—a situation which is being reinforced by deteriorating health (sub-category*“It Limits Us in Many Ways”: Transitioning Towards Uncertain States of Chronic Illness and Disability*) and their inability to independently navigate formal support services without the help of their personal support network (sub-category*“Asking for Assistance Is Difficult”: Navigating a Volatile and Unpredictable Welfare and Healthcare System*).

#### “Asking for Assistance Is Difficult”: Navigating a Volatile and Unpredictable Welfare and Healthcare System

A third contextual factor building a “welt of insecurity” around our respondents was situated at the structural level, namely the volatile and unpredictable formal support from the local or national government in form of social welfare provision, available pension schemes, and particularly access and affordability of healthcare services. Residing in rural areas and being highly dependent on agricultural incomes, many respondents talked about their difficult economic situations. After retirement, only very few people were entitled to an official pension and thus had to continue making a living somehow: “*I didn*’*t get any pension after retiring from my previous job because I was not a civil servant. Now my daily job is opening a kiosk.*” (Woman from Karangmojo, FGD 7). Those respondents who are receiving a pension, reported about the unreliable payment schemes and unfulfilled promises: “*Last year there was an increase in salary ( = pension), plus a 13th salary. But this year there is no increase. They said there will be a 13th and 14th salary instead but it didn*’*t come*” (Woman from Semanu, FGD 5). While there has traditionally always been patchy formal economic support, this situation is aggravated by the increasingly expiring informal economic support from their children and their declining health limiting their ability to work.

However, the most commonly discussed struggle reported by our respondents pertained to their dealing with the healthcare system—an issue which is clearly overlapping and reinforced by health-related uncertainty (sub-category *“It Limits Us in Many Ways”: Transitioning Towards Uncertain States of Chronic Illness and Disability*) and eroding intergenerational cont(r)acts (sub-category *“We Are Left Behind”: Sociocultural Change Eroding Stable Intergenerational Cont(r)acts*). In 2011, a new Social Security Administration Board Law (abbreviated in Indonesian as BPJS) was passed which aimed at synchronizing fragmented healthcare provisions and insurance schemes. Additionally, in 2014, a system for the implementation of Universal Health Coverage (UHC) was implemented with the aim to cover 100% of Indonesians by 2019. In 2016, the year this study was conducted, respondents reported several hurdles they were facing amid the implementation of this new healthcare reform. For instance, the most commonly reported issue pertained to a lack in transparency about which facilities and services they could access for free and which needed to be co-paid or completely paid out of pocket. Also, which diseases and medications were covered by the new scheme remained unclear to them and they were critical towards the newly implemented “*complicated referral systems*” (Woman from Wonosari, FGD 3). While it goes beyond the scope of this study to go into further details around the implementation of BPJS and UHC in this rural setting, these examples are again helpful in understanding how strongly the respondents were depending on their children to help them navigate in this new healthcare arena, as exemplified by two respondent: “*I have a heart disease and the medicines are very expensive. (…) I asked my son to go and apply (for BPJS coverage) for me. To get assistance from the government is very difficult. It*’*s been four months and it*’*s not approved yet.*” (Woman from Karangmojo, FGD 7). Yet another respondent told that “*eventually, I didn*’*t go to the hospital to see a doctor. (…) there are many administration requirements and we needed to copy this and that…then my son lost his patience because he had to take me here and there.*” (Man from Semanu, FGD 6).

By unfolding health-related, social network-related and government-related sources for rising insecurity in respondents’ aging process, the above has outlined the micro-, meso-, and macro-level contextual circumstances of this study. The frequent comparison between past and present and between themselves and younger (and older) generations was key in unfolding these three distinct but also clearly interconnected and reinforcing contexts. For example, throughout their lifetime (most were born during the 1950s) respondents have always struggled with unreliable formal welfare protection and inadequate healthcare provision, particularly considering that respondents lived through major social and economic crises and reforms during the past decades. However, from their accounts, a stable and reliable intergenerational contract has in most cases worked as a buffer for poor formal protection. Likewise has this contract worked as an insurance in old age when their health and function declined. However, respondents declare that *“this era […] has passed”* (Man from Wonosari, FGD 4) and eroding intergenerational solidarity, diminished observance of filial piety and diverging interests between generations do not buffer anymore health or economic hardships. Constantly occurring states of insecurity from health crises, sociocultural changes, and volatile government support have not only fundamentally disrupted the institutionalized logic of intergenerational old-age care but also transformed the process and perception of aging and the aged. Instead of spending out their lives within the close family circle and being a respected and well-cared for elderly, respondents pictured themselves as being “*outdated*” and “*being left behind*”. From our data we see that this risky aging process of our respondents is less framed by their individual inherited vulnerabilities stemming from their advanced age, sex/gender, socioeconomic position, or the inherent challenges in this rural setting. Instead, our respondents appear to be aging surrounded by what we term a “welt of insecurity”, i.e., a lifeworld characterized by permanent states of insecurity which have been socially produced by recent epidemiological transitions and a context of declining or deficient informal and formal networks of support.

### Older Adult Children: A Generation “Betwixt and Between” Expected Demands and Unmet Expectations (*Cause*)

This second category is describing the causal condition from which the core phenomenon and the other categories emerged from. While the previous category (*context*) has outlined contextual explanations why and their own aging process transformed into an insecure trajectory, this category (*cause*) delineates how this “welt of insecurity” has impacted the identities and roles of our aging respondents. “*Older Adult Children: A Generation “Betwixt and Between” Expected Demands and Unmet Expectations*” describes the multiple and contradictory landscapes of our respondents’ older adulthood and the liminal space that they inhabit in-between these two landscapes.

From our FGDs it became obvious that the respondents were adult children and aging parents simultaneously. Oscillating between care-giving demands from their elderly parents and care-receiving expectations towards their middle-aged children, their older adulthood is occupied with two inconsistent identities and roles which are based on increasingly contradictory and conflicting sociocultural norms, values and beliefs systems.

In our data, both the contrasting notions of continuity and discontinuity together with the permeating impression of being in a liminal in-between space and place appeared to be central in unfolding the *cause* in this study. Respondents’ threatened identity, their inter-role conflict, as well as their emotional ambivalence between feelings of continuity and discontinuity are constructed from the two polyphonic sub-categories, “‘*It Is Our Duty’—Remaining a Respectful Child”* and “‘*Let’s just hope for the best’: Fearing to Become a Neglected Parent”*.

#### “It Is Our Duty”: Remaining a Respectful Child

If it is the integration of opposites that defines the ambiguous identity of liminal personae, then the “respectful child” represents respondents’ previous identity rooted in the familiar and traditional past. Accounts of this identity are characterized by clear and structured language and were frequently re-iterated during the discussions. Respondents provide a continuous and legitimate narrative of the respectful and dutiful child. Such children show commitment and determination in fulfilling their caregiving duties to their parents. Caregiving duties are an internalized unquestioned quality of their identity: “*Taking care of our parents is not just an obligation, it is within ourselves*” (Man from Semanu, FGD 2). Religious, spiritual and cultural frames are further legitimizing this obligation and their dutiful identity: “*We must honor our parents. It is an obligation according to our religion, any religion*.” (Man from Wonosari, FGD 4) and “*(…) it would be a sin in Javanese culture if we don*’*t (take care of our parents)*” (Woman from Semanu, FGD 1). The identities of being a “good daughter” and “good son” do not come easily but the accompanied burden is viewed as a means of personal and sometimes spiritual fulfilment.

Being and remaining a dutiful and respectful child is also a carefully normatively and culturally guided experience. From our data, it was obvious that the overall village community—but most commonly their same-aged peers—scrutinize these good daughters and good sons ensuring the fulfilment of their filial duties. Those who are not fulfilling their filial duties were questioned by the community and both the children and parents became subject to gossip, shame, embarrassment, stigma and social exclusion: “*(…) Yes, there will the gossip about that. (…) People are like this. In our deepest heart, we cannot accept it (…) because of our principles. Especially daughters…we want them to pay back their parents’ deeds. In Javanese, we call it* “*ora mentolo*”*. We really feel pity.*” (Man from Wonosari, FGD 4).

Taking on the role as their parents’ caregivers is a logical and anticipated step in their lives. It’s an institutionalized obligatory role for each child and happens in a relatively well-defined time frame: “*We were taken care of by our parents from pregnancy until we got married. (…) My parents took care of me longer than I (will) take care of them.*” (Woman from Semanu, FGD 5). The role of a caregiver is a clearly defined and socially accepted and anticipated role, however, respondents also expressed concerns about their bodily capabilities due to their older age and already declining health status. The phrase “*we are old already*” was commonly used as an explanation and apology for any shortcomings in their caregiving tasks. Compared to the last generation, taking on the roles as caregivers happened later in their lives than they expected and when they themselves are already dealing with chronic health problems.

Respondents’ emotions with regards to their role as adult children appeared positive, i.e., they express motivation to take on the role and fulfil their duties, it gives them fulfilment and a sense of self-efficacy. Because they make it possible for their own parents to “*grow old normally*” (Man from Wonosari, FGD 4), a sense of pride was also a commonly expressed feeling. Any stress or burden is expressed as manageable as they know this period in their life is only temporary. Much motivation also comes from their hopes to receive the same standard of care from their own children later, as one women expressed: “*We hope it will be the same*” (Woman from Karangmojo, FGD 7). However, many perceive this as unrealistic as one respondent states: “*(we) will see that (our) hope will not be fulfilled*” (Man from Wonosari, FGD 4). Remaining dutiful and respectful children against the odds of chronic insecurity surrounding them, provided them with feelings of stability and order. They continued to live by the rules of the past intergenerational contract which creates a sense of both biographical and sociocultural continuity for both themselves and their elderly parents.

#### “Let’s Just Hope for the Best”: Fearing to Become a Neglected Parent

While the “respectful child” occupied one side of our respondents’ older adulthood, the “neglected parent”, or the fear to become one, stood on the other side. In-between stands a liminal generation of OACs who is departing from a secure context towards insecure life spaces. Respondents expressed a fear of a discontinuing past including their prescribed role in it and they also had very little clarity about how their new identities or roles will shape. Many respondents identified themselves as an older and sick parent (i.e., an eligible care-receiver) but also expressed that this identity was not granted to them as their children did not anymore follow the past intergenerational contract. At the same time this conflicted with their struggle to remain respectful children’ and care-givers to their elderly parents. Respondents struggled to find a legitimate narrative for their identity of “neglected parents” as this is not a logical or anticipated step and has no normative guidance or reference frame.

This lack of normative guidance was exemplified by many untraditional make-shift solutions which reified their feelings of disorientation and ambivalence with their fear of being or becoming a neglected parent. This was most clearly expressed in economic transfers between generations. While economic transfers were traditionally upwards from younger to older generations, in our data, we found many instances where respondents reported that they asked financial assistance from their elderly parents, i.e., a downward transfer, or where one or even two generations were skipped, i.e., support from grandchildren to respondents or from respondents to respondents’ parents, as one women tells: “*None of my children sends me any money. (…) But I*’*m sick and I need to eat. So, I went to my father*’*s house and he gave me some [money].*” (Woman from Wonosari, FGD 3).

In our data, we observed that feelings of disorientation and discontinuity were channeled through two emotions. One was characterized by the Javanese *nrimo* philosophy, i.e., passively observing and understanding whatever is happening and being content with it. This mood emerged from frequent use of (translated) phrases like “*Let*’*s see.*“, “*Let*’*s hope.*“, “*We don*’*t want to become a burden.*” and “*We have understood the consequences.*“. The other was characterized by what we term a mood of subjunctivity, a language of doubt but also of hope and for seeing potential in the new. Through subjunctivity, respondents used the state of liminality in a positive way by carving out spaces and situations in which this blurry situation became a resource, personally and socially. It enabled them to visualize alternative futures, potentials and possibilities; particularly men saw it as an encouragement for personal and spiritual growth, i.e., passing a test: “*That is called puluh-puluh. When you can get through it, then you pass your test*.” (Man from Semanu, FGD 6). Many expressed also hope that the government in the future will provide more help in supporting older people: “*I do hope, indeed, but I think it*’*s still difficult.*” (Woman from Karangmojo, FGD 7).

### “The Devil’s Company”: Landscapes of Loneliness (*Consequences*)

This third category deals with loneliness and is as such describing the emotional consequences of aging in chronic insecurity (*context*) and holding a liminal position of an OAC (*cause*). From our data, loneliness manifested on multiple levels and was emerging through multiple pathways. On one hand, loneliness was described and experienced as an individual and social phenomenon; on the other hand, this emotion was both a consequence of their insecure and liminal circumstances and simultaneously a consequence of taking unconventional actions to alleviate insecurity and liminality. These aspects are described in the two sub-categories *“‘There is No Medicine for Such a Pain’: Loneliness as Corporeal Suffering”* and *“‘I Was Not a Human Anymore’: Loneliness as a Social Stigma”*. Taken together, these two sub-categories display both individual and social entities of loneliness, which we subsume here as the various “landscapes of loneliness”. Juxtaposing these two categories shows that loneliness was perceived both in its traditional sense with a strong relational (or lack thereof) focus and individual-level physical and mental consequences but also from a perspective that underlines how tightly this emotion was interwoven with the fabrics of OACs’ social contexts and as such stood out as a defining aspect of their aging process. What was likewise conveyed is how these different ways of framing loneliness strongly impacted the way respondents could adjust their actions. These action strategies are detailed in *“For Sure, There Will Be Gossip”: Compromising Against Conventions (Actions)*.

#### “There Is No Medicine for Such a Pain”: Loneliness as Corporeal Suffering

This sub-category outlines how the emotion of loneliness appears as an individual-level phenomenon. In our data, this conceptualization was mainly driven by the emergence of codes relating to the physiological and mental processes relating to loneliness. The corporeal, i.e., physical and mental aspects of loneliness were one of the most frequently discussed topics in the FGDs. From this large amount of data, we could distinguish between three perspectives which describe respondents’ subjective negative understanding and meanings of loneliness.

First, loneliness was understood as being a disease itself and as such causing both bodily and mental symptoms. Second, loneliness was believed to cause disease, and third, the reverse, that certain diseases cause loneliness. As exemplified by the following quote, particularly chronic conditions that affect mobility and sensory impairments (particularly hearing) were mainly believed to be causing a feeling of loneliness: “*As long as you can move and communicate, it will not limit you. But those who are always ill, they will be lonely.*” (Woman from Wonosari, FGD 3). Another respondent explained a reverse pathway, i.e., how long-term loneliness causes disease, like he noted: “*Prolonged loneliness means it ( = loneliness) is deeply buried in your heart. It will create a weakness in your body and you turn ill.*” (Man from Semanu, FGD 6). Probing for detailed examples showed that in fact their understanding appeared to be more nuanced with regards to the positioning of certain types of diseases within this process. Respondent ultimately described a pathway of how physical health problems caused loneliness and that loneliness subsequently led to mental health problems. Particularly perceptions about the relationship between loneliness and mental health appeared to be very nuanced among respondents. They most commonly used expressions such as “*a wandering mind*”, “*an empty mind*”, “*daydreaming*”, or the idea of “*thinking too much*” to describe the mental health implications among the lonely.

However, irrespective of whether loneliness was perceived as a disease itself, or being the cause or effect of it, respondents uniformly agreed that “*there is no medicine for such a pain*” (Woman from Semanu, FGD 1). We see this quote (or the cluster of codes this quote represents) as central for shedding light on two closely interconnected issues, namely the understanding of the pathways leading up to loneliness and the possible range of actions to address this negative emotion. First, framing loneliness as a disease and admitting the absence of a “medicine” puts the responsibility of curing it - or taking actions against it - on the individual. Based on our data, a pathway from the contextual and causal factors to loneliness became apparent. The combination or clustering of chronic health problems (sub-category *“It Limits Us in Many Ways”: Transitioning Towards Uncertain States of Chronic Illness and Disability*) in an environment of eroding formal (sub-category *“Asking for Assistance is Difficult”: Navigating a Volatile and Unpredictable Welfare and Healthcare System*) and informal support networks (sub-category*“We Are Left Behind”: Sociocultural Change Eroding Stable Intergenerational Cont(r)acts*) resulted in the feeling that health and social needs are increasingly not being adequately met by one’s social or care networks and resulted in the emergence of loneliness among these rural OACs. Respondents provided several examples of individual actions to alleviate the feeling of loneliness. “*Distracting the mind*” with watching TV was described as way to cope with loneliness: “*It is difficult to be lonely in these modern times…we have a TV. That can fill our minds.*” (Woman from Karangmojo, FGD 7). For men, getting out of the house and socialize was key in dealing with feelings of loneliness. This strategy was however highly guided by gendered and cultural norms as one respondent explains: “*Me as a man -I*’*m sorry to say this- but I am freer. I can go anywhere and socialize with my friends. Yet for women, they cannot. (…) Especially if a widow wants to go out alone…it will be very suspicious.”* (Man from Karangmojo, FGD 8). As such, gender and marital status frame both the vulnerability of becoming lonely as well as the available action strategies to cope with it.

#### “I Was Not a Human Anymore”: Loneliness as Social Stigma

This sub-category describes the social spheres of loneliness. It is doing so by unfolding how loneliness emerged in response to social exclusion. We further describe how these exclusionary experiences have led to the surfacing of new group goals and norms among OACs, and subsequently to the adaptation of unconventional actions (*“For Sure, There Will Be Gossip”: Compromising Against Conventions*). Ultimately, from the accounts of the respondents it appeared that loneliness likened a social stigma because of lacking the emotional fit with the prevailing sociocultural norms.

As outlined earlier, OACs stood out as a liminal group oscillating between the roles of adult children and neglected parents. With descriptions such as “*outdated*”, “*old-fashioned*” and “*left-behind*”, OACs repeatedly describe themselves by differentiating between them and other groups. Another defining feature of OACs is their exclusion from different social systems. In our data, we found frequent economic, religious, and social media/technology related explanations for loneliness among OACs. From an economic perspective, loneliness is a consequence of families and the government not providing sufficient economic support for OACs. In our data, money is pictured as a vital means to join social activities such as the monthly *arisan* in the village. In this sense, money is a way to buy togetherness. A volatile welfare system and children increasingly investing less in their parents has put many respondents into a difficult economic situation and has strongly impacted their opportunities to socialize outside their own household: “*Old people feel lonely when they don*’*t have any money. (…) If my children don*’*t send money to me, then I cannot join social activities. Everybody would know that my children didn*’*t send money to me. I feel ashamed and stay home.*” (Woman from Wonosari, FGD 3). From a religious perspective, loneliness is a consequence of lacking a connection to God and lonely people are thus more inclined to socialize with the devil: “*If you*’*re lonely, only the devil will accompany you.*” (Man from Wonosari, FGD 4). Following this argumentation, the only possible solution to end loneliness is getting closer to God. Praying, reading the scripture and attending services was a very common way of our respondents to deal with their loneliness. Another system which is excluding older people from participating and interacting in society is related to social media/technology. Respondents repeatedly explain how they are limited in connecting with the younger generations because they are not able to follow their modern topics (see *“We Are Left Behind”: Sociocultural Change Eroding Stable Intergenerational Cont(r)acts*) or because they are not able to use modern technology (e.g., smartphones): “*We only have the old cheap phones. Only for making phone calls*.” (Woman from Wonosari, FGD 3).

In sum, these three perspectives, outline that loneliness unfolds as a social problem in Indonesia despite its seemingly individual and subjective experience. Loneliness appears to be a consequence of different social systems excluding older people in different ways: being systematically excluded from religious context, having restricted access to financial means to join social gatherings, and to losing a voice to engage and communicate. Such experiences were not voiced in any physical or mental suffering but on an existential level as expressed by one man “*I was abandoned. (…) left behind, like I was not a human anymore.*” (Man from Wonosari, FGD 4). Our data showed that the exclusion of OACs from certain social systems shaped their perception of loneliness not only as a defining issue for their group but also as a reason for developing unconventional actions (*“For Sure, There Will Be Gossip”: Compromising Against Conventions*).

Since the situation of OACs clearly differs from other groups, their expressions of loneliness can be considered as being not in concordance with the prevailing norms. Loneliness is framed as a social emotion which is emerging as a consequence of OACs’ aging process and as such an emotion which is systematically shaped by the social-contextual influences, i.e., the chronification of insecurity and liminal positioning of OACs. Loneliness as a result of OACs aging process directed respondents to adjust their actions. As opposed to the individual-level framing of loneliness (*“It Limits Us in Many Ways”: Transitioning Towards Uncertain States of Chronic Illness and Disability*) these actions are collectively adopted in order to take collective action. The quote “*What can we do? We need to adjust ourselves.*” (Woman from Semanu, FGD 1) summarizes that within the group of OACs, new norms have been formed with the common goal to re-integrate into social systems and work against exclusion. Loneliness is a consequence of their specific aging process and as such directed OACs to adopt specific actions (*“For Sure, There Will Be Gossip”: Compromising Against Conventions*).

### “For Sure, There Will Be Gossip”: Compromising Against Conventions (*Actions*)

This category outlines the action strategies taken by our respondents to react to and overcome the threats of chronic insecurity and liminality. Respondents provided numerous examples of how old-age care was traditionally arranged (e.g., by describing how they provide care to their elderly parents) and how care is negotiated in modern times (e.g., through their narratives describing their fears of becoming neglected parents). Respondents clearly identified an “ideal case” and a “worst scenario” of how old-age care may take shape and place. Between these two extremes, in our data we discovered various adaptations–or unconventional compromises–that have been gradually adapted in order to cope and make their risky positioning at least temporarily more stable and secure—both in terms of securing care and alleviating loneliness.

The ideal case was described as a form of joint inter- and intra-generational network of close-kin carers such as spouses, children, grandchildren or siblings. The description of such a “circulation of care” where different family members, but primarily children, and the ideas of “*taking turns*” was dominant across all FGDs and described not only the optimal care model with the best quality but also allowed care to take place in the care-recipients’ homes. The most commonly cited example of a joint circulation of care would be a wife who shares the caretaking responsibilities to her husband together with her children, with occasional involvement of grandchildren or daughters-in-law. This system of “*taking turns*” promoted shared decision making and was also a way to mitigate the care burdens among the carers, which likewise contributed to keeping the quality of care high. However, with increasing frequency, respondents noted that disharmony among the different generations increased because the younger generations are less willing to assume their traditional roles and responsibilities. Social change as the main driver of these eroding intergenerational contracts has been described in *“We Are Left Behind”: Sociocultural Change Eroding Stable Intergenerational Cont(r)acts*.

Being institutionalized into an elderly care facility outside the home village, however, was undoubtedly described as the “worst case scenario” both in terms of quality and the preferred place of care. It was described as a “*shameful act*” (Woman from Karangmojo, FGD 7) or an “*abandonment*” (Man from Karangmojo, FGD 8) and while many agreed that the use of care facilities was something that occurred mostly among the “*city people*” (Man from Karangmojo, FGD 8), they also realized that such “unconventional” care arrangements may increase also in their rural villages in the future, as one notes, “*It*’*s not yet here*” (Woman from Karangmojo, FGD 7).

Based on the accounts of our respondents, in-between these two extremes occurred three “unconventional compromises”: the more frequent occurrence of intra-generational or older-to older-care (e.g., between spouses or same-aged siblings), the increasing involvement of distant or non-kin carers (e.g., nieces, nephews, or adopted caretakers), and the emergence of “care communities” (e.g., care arrangements between neighbors). The following two sub-categories *“‘But It Doesn’t Feel Comfortable’: Compromising on Quality of Care”* and *“From “Home Care’ to ‘Care Home’: Spatial Compromises in Old-Age Care”* outline in which ways compromises in terms of quality and place were made and in how far these were viewed as going against the traditional conventions but served as a means for OACs to get a “sense of security” -both in terms of care provision and alleviation of loneliness.

#### “But It Doesn*’*t Feel Comfortable”: Compromising on Quality of Care

As outlined in *“We Are Left Behind”: Sociocultural Change Eroding Stable Intergenerational Cont(r)acts*, the norms of filial piety, kinship obligations and various religious or normative commands create the deeply imbedded expectation that children obtain the prime duties to take care of their aging parents. These expectations are also highly gendered as the main responsibilities fall upon daughters and wives. However, children have become an uncertain source of support and when the old intergenerational contract is not adhered to, compromises, often against conventions, must be made. All three compromise solutions which we identified from the data are characterized by a decreasing amount of “quality” in care. While these three strategies are presented separately, they oftentimes interact or complement each other, i.e., actions are not mutually exclusive but respondents would at different times utilize different actions to secure a temporary secure situation.

If children fail to provide care, the first best option to compensate for such deficits was to rely on a care network consisting of one’s next-of-kin within the same generation. Such intragenerational older-to-older care, i.e., between spouses or same-sex siblings represented a lower quality of care because it clearly conveys the picture that children did not assume their caretaking obligations towards their aging parents. Especially a husband taking care of his wife goes against the prevailing normative and gendered norms that dictate old-age care in our respondents’ settings. Still, despite the obvious re-traditionalization of family bonds and in some-cases de-traditionalization of gender relations (i.e., when husbands are their wives’ caretakers), such arrangements—despite being unconventional—allowed them to create a “sense of security” into their care-arrangements.

Quality of care was further reduced when relying solely on a care network of more distant family members, such as nieces or nephews or by adopting a caretaker into the family. Such arrangements more clearly signify the “*abandonment*” by one’s own children. Widowhood—or the anticipation of it, created a strong sense of uncertainty and insecurity among respondents, as one man noted, “*If my wife dies first, I have no one who takes care of me*” (Man from Semanu, FGD 2). While relying on distant kin ensured a moderate security in relation to old-age care but respondents also admitted their limitations: “*They (distant-kin carers) do help of course, but not fulltime*” (Woman from Wonosari, FGD 3).

The widest extension of care outside the kinship network, and with this the lowest quality of care, is exemplified in the third strategy, which considers the involvement of neighbors or the wider community, yet for restricted aspects of care. Compared to the previous strategy, the list of tasks one could enquire from such a network is further reduced. What has become clear from the accounts of the respondents is that such community networks are not at all involved in any personal care or in providing any financial support—but they provide mainly everyday helps or emotional support. From the accounts of the respondents, it showed that the involvement in such caring communities is also highly dependent on one’s socioeconomic and health status. The concept of reciprocity guides the involvement in such community networks. For example, a good degree of physical functioning is required in order to socialize in these networks, as one woman notes “*You don*’*t want become a nuisance*” (Woman from Karangmojo, FGD 7). Further, many activities involve the contribution of small amounts of money (e.g., collection of social funds, donations or participation in the *arisan*). Old-age care from the perspective of these community networks is perceived as a burden which should be shouldered collectively–something which is expressed in Indonesia through the *gotong royong* philosophy. The “*spirit of gotong royong*” was discussed in many FGDs as expressed by one man: “*We are a good community, the spirit of togetherness is still strong*” (Man from Karangmojo, FGD 8). However, these village networks would also step in when children fail to provide support, which sometimes would result in conflicts between the village networks and the children as noted by one respondent: “*How can we offer support if they don*’*t want to be supported?*” (Man from Semanu, FGD 6).

#### From “Home Care” to “Care Home”: Spatial Compromises in Securing Care

Spatial compromises were less commonly discussed but still stood out as a defining aspect of OACs unconventional action strategies. While quality of care was strongly related to the positioning of carers in respondents’ social networks (e.g., centrally positioned close kin vs peripheral ties outside the family) and the assignment of caretaking tasks, spatial compromises reflect the degree to which old-age care has been moved outside the home of the care-receiver. The stark opposition between the home space in the “ideal scenario” and the institutionalized placing outside the village were the initial starting point to look for nuances in terms of similarities or opposites between these two scenarios. The quote “*Moving to a child*’*s house can sometimes be a very sad experience*” (Man from Semanu, FGD 2) led us to also pay attention to the dimensions of agency while making such spatial compromises. This was crucial in understanding whether respondents viewed such spatial changes as conditions for opportunity or limitation.

While intragenerational elder-to-elder care was more compromising in terms of quality of care, the spatial compromises were less strong because most intragenerational care arrangements continued to take place in respondents’ homes. The second compromise solution, i.e., involving distant or non-kin carers likewise -while being highly controversial and stigmatizing in terms of quality of care, again the actual care work continued to take place in the own home but a distant carers would oftentimes move into the home of the care-receiver. In this sense, lower quality care is restricted to the home spheres. The third strategy entails the involvement of community networks and in this sense “moves” aspects of old-age care outside the private home into the public village spheres. With the ultimate extension of care outside the home space, respondents actively created opportunity loci and new social spheres for maintaining or extending their care networks. This stands in contrast to the previous strategy where the decision was made to extend care networks within the own home.

### “Bargaining for a Sense of Security”—A Grounded Theory How OACs Experience Their Aging (*Core Category*)

Abducting from the accounts of 48 OACs, four categories emerged: 1) “Aging in a Welt of Insecurity” (context); 2) “OACs: A Generation ‘Betwixt and Between’ Expected Demands and Unmet Expectations” (cause); 3) “Landscapes of Loneliness" (consequence); and 4) “Compromising Against Conventions” (actions). The core phenomenon of this study appeared to be the social practice of “bargaining for a sense of security” which essentially describes a process by which OACs experience their own aging. The core phenomenon reflects respondents’ goals to transfer their aging process from states of chronic insecurity to a temporary sense of security. The conceptual model (Figure 3) reveals the relationships among the four categories and their relation to the core category. Although the occurrence of this process appeared quite homogeneously among our respondents, a number of intervening conditions influenced respondents’ process of “bargaining for a sense of security”. Such intervening conditions are represented in terms of vulnerabilities with regards to respondents’ sex/gender, socioeconomic position, social network capabilities, health status, and the rural context influence.

## Discussion

The present study proposes a Grounded Theory and conceptual model of how OACs experienced their own aging as a process of “bargaining for a sense of security” and by this provides valuable insights into how and why loneliness emerged as both a health and social problem amidst the changing realities of social and demographic transformations in rural Indonesia. This study is as such one among few to report on loneliness among Indonesian populations (Peltzer and Pengpid, 2019) and the first to explore this phenomenon from a qualitative social network perspective. During the reporting of the results, we have made efforts to interpret already various aspects of our findings and put them into the wider Indonesian context. The following discussion centers on two selected overarching issues: first, the core phenomenon, its relation to the social construction of risk and the framing of OACs’ aging process as a process characterized by chronic insecurity; and second, the social dimensions of emotions, their capacities to affect social life and in this sense how loneliness has been theorized as a driver to alter the norms around traditional care arrangements in our study.

As outlined in the method section, the social construction of risk guided our theoretical approach to the data (Luhmann, 2005; Arnoldi, 2009; Burgess et al., 2014). As such, the core category “bargaining for a sense of security” represents the flipside to the risk construction approach, i.e., it resembles the social construction of security as opposed to risk. The core phenomenon centering around the importance of “security” mirrors insights from anthropological and ethnographic work which approaches “security” from a social practice perspective. For example, as von Benda-Beckman described it, security as a social practice represents attempts to overcome insecurities related to one’s existence, which may include securing basic commodities like water, food, shelter, income and health. Some studies, including our study, added “care” as one such commodity (von Benda-Beckmann and von Benda-Beckmann, 1994). In our study, experiences of insecure care manifested on different levels highlighting both informal and formal care insecurities on the meso and macro levels. As such, contextual markers of insufficiency, inadequacy, and low-quality relating to informal (mostly family-based) care networks and the formal support from the healthcare system stood in close relation to the consequent emergence of loneliness. These insights emphasize the importance to address old-age loneliness through multi-systemic approaches and interventions that aim to integrate formal and informal networks to promote health and wellbeing of aging populations and their aging caretakers.

In addition to “insecurity”, our study adds the notion of “chronification” to it. Our conceptualization of the context in which OACs are aging—“a welt of chronic insecurity”—shares similarities with other studies and how they conceptualize the experiences and contexts of population aging. For example, Van Eeuwijk and colleagues, propose the “triangle of uncertainty” to describe the socioeconomic and health-related contextualities in which older adults in urban Sulawesi and Tanzania are growing old in (van Eeuwijk, 2006; van Eeuwijk et al., 2016; van Eeuwijk, 2020). Our work also shows interesting connections to anthropological work on chronic diseases through the lenses of chronicity and syndemic suffering (Kleinman et al., 2010; Weaver and Mendenhall, 2014; Manderson and Warren, 2016). Traditionally, risks for a poor health outcome have been articulated in terms of vulnerabilities of certain sub-populations, i.e., theorizing potentiality for a negative outcome (Schröder-Butterfill and Marianti, 2006). In this sense, our conceptualization of insecurity relates to potentiality. However, we add to a more nuanced understanding of old-age vulnerability by adding the idea of “chronification” which is essentially driven by demographic and social change, earlier referred to as “megatrends”. Particularly the eroding intergenerational contract exemplifies how such megatrends have replaced perceptions of structured security with chronic insecurity. The focus on the concept of “bargaining for a sense of security” also insinuates that in the context of such megatrends and processes where insecurity becomes a chronic state, only a “sense” of security can be achieved but not by the avoidance of threats but the temporary management of risks.

Second, theorizing the interpersonal emotion of loneliness as a driver to alter traditional old-age care arrangements, particularly networks, has been a key insight from our study. Specifically, loneliness affected OACs action strategies for securing care and as such altered, within this group, the traditional norms and expectations surrounding old-age care. Loneliness emerged as the consequence of a process which describes aging as a “bargaining process for a sense of security”. We described the social aspects of old-age loneliness which stand in close relation to a set of “unconventional actions” aiming at alleviating loneliness and securing old-age care. As such, our findings are one of the first to highlight the social dimensions of loneliness and this emotion’s capacity to affect social life and norms. While many have highlighted the growing awareness of emotions’ intrinsic link with social life, studies addressing such questions on a group or cultural-level are considerably underrepresented (Keltner and Haidt, 1999; Schirmer and Michailakis, 2016; Van Kleef, 2016). To the best of our understanding, the majority of studies on loneliness apply a medical or psychological perspective emphasizing on the associations of loneliness with physical or mental health. This strong focus on delineating this emotion as a medical problem has been recently rendered in discussions around the over-medicalization of loneliness (McLennan and Ulijaszek, 2018). To the best of our knowledge, only few address the social aspects of loneliness or approach the condition as a social rather than a health problem. For example, a study by Schirmer reifies how the systematic exclusion from social function systems relates to loneliness (Schirmer and Michailakis, 2016). However, we are unaware of any studies theorizing on this emotion’s capacity to affect social life or social change. Over the past decades, scholarly awareness on emotions’ capacity to influence people and their norms has increased in the area of emotion research, but emotions’ role in public health research remains scarce (Van Kleef, 2016).

Taken together, our study described a process where multiple negative experiences intersect and as such constrain and negatively shape the aging experiences of our rural Indonesian respondents. This study made several attempts to theorize the social construction of risks by reifying how chronic insecure contexts cause the liminal positioning of OACs and consequently lead to stigmatizing feelings of loneliness which are bidirectionally related to a set of socially less-accepted “unconventional” actions which compromise quality of old-age care. The intersections of these multiple negative experiences resulted in a picture which has recently been subsumed under the “precarity” terminology and which extends the focus from inherited or traditional forms of vulnerability to modern or produced forms of precarity (Rigg et al., 2016; Cruz-Del Rosario and Rigg, 2019). For example, in our data it became obvious that rurality or low socio-economic context have traditionally made older adults more vulnerable to adverse health or wellbeing outcomes while social networks acted as essential safety nets to buffer these negative effects. However, the eroding effect of social change and population aging on social networks—particularly informal care networks—produced new forms of vulnerability, i.e., precarity. Precarity is an emerging concept in aging studies but remains scarcely explored in LMIC settings. Many have used the concept of precarity to theorize processes of modern social and economic transformations, particularly for understanding the insecurities and risks experiences by older people (Grenier et al., 2020). More recently, a framework for precarity and health was proposed based on research on various material-need insecurities among older United States women with (or at risk for) HIV (Whittle et al., 2020). Our findings show some interesting overlap, particularly with regards to aspects of experiencing uncertainty, stigmatization and social acceptability. Further, our results enable a conceptualization of the notion of precarity on different levels spanning micro, meso and macro levels. Particularly narrations of care burden and role conflicts among OACs exemplified how precarity was socially constructed within the interactions and interdependencies in informal social networks. To the best of our knowledge, our study is one of the first to incorporate how precarity and loneliness interrelate in the lifeworld of OACs in a transitioning LMIC context. The pathways through which social networks may exacerbate precarious aging trajectories and negatively impact health and wellbeing among aging Indonesian populations have been explored in our previous work on the health effects of social network diversity (Schröders et al., 2020) and the role of network dynamics on healthcare utilization outcomes (Schröders et al., 2021).

### Strengths and Limitations

A key strength of this study is that it is among the first to address OACs. This population segment is not an unusual dynamic anymore in LMICs and clearly presents new demands for old-age care provision. Besides this, this study has updated the field of social network and aging research in Indonesia into the area of old-age and long-term care and is among the few to provide evidence on loneliness as an evolving public health problem among older Indonesians. In this sense, our findings do not only add to the advancement of theoretical knowledge but the results also in several ways have implications for building more “secure”, i.e., sustainable and equitable old-age care policies and systems in Indonesia and similar settings. Above we already presented in which ways our study met the trustworthiness criteria as outlined by Lincoln and Guba. We believe these share many similarities and overlap with the two criteria for the assessment of Grounded Theory studies as outlined by Strauss and Corbin (Strauss and Corbin, 1998; Corbin and Strauss, 2014). These relate to 1) judging the adequacy of the research process, and to 2) assessing the empirical grounding of the findings. The theoretical sampling frame and the dense descriptions (e.g., of the data collection process, the demographic and epidemiological aspects of the study settings, emergent designs) aided the evaluation of the first criterion. Both the application of the consequential matrix, which led to more density in terms of properties and dimensions of the categories, and the constant comparative method, which further verified the consistency of emerging concepts, strengthened the empirical grounding of the findings.

A commonly shared limitation of Grounded Theory studies with emergent analytical goals, is that it is not possible to make generalizations (in a statistical sense). Additionally, being set in a small geographic area with little ethnic diversity, the transferability of our finding to other non-Javanese contexts may be reduced. We aimed to provide a detailed description of the setting and the context to that readers are able to judge to which degree our results may theoretically appropriate to other contexts.

Two limitations may derive from the cultural and linguistic barriers of the first author who took the main responsibility during the data collection and analysis. This was particularly visible from two issues. First, despite using a modified interpreter-assisted approach, the course of the FGDs was mainly steered by the native fieldwork assistant and to a lesser degree by the first author. While this may bring about limitations, we also see this as a strength of our study as we were able to combine “insider” with “outsider” perspectives during different phases of the study. We argue that this enabled us to both remain culturally sensitive perspective when approaching the data as well as to be able to detect facets of the data which may have otherwise been hidden by a “blinder of familiarity”. The second issue pertains to the cross-language process of collecting and analyzing the data. While data collection was conducted in Indonesian/Javanese, the analysis was conducted by the first author based on the English-language transcripts. Many have argued that this cross-language process of data collection and analysis can pose serious threats to the trustworthiness of a qualitative data (Squires, 2009; Squires et al., 2020). Particularly transcriptions and translations provided by persons not involved in the research can introduce interpreter-mediated vulnerability into a study since their quality will affect the entire analysis and interpretation process (Squires, 2009; Squires et al., 2020). Particularly culture-bound meanings, connotations of words, or proverbs may get lost in translation. We tried to limit these threats in two ways. First, we extended the role of the interpreter from a functionalist to an interactionist role. Specifically, this means that we did not only rely on the interpreter’s transcription and translation of the data but we utilized the interpreter as a cultural and linguistic broker and thereby contributing to culturally appropriate explanation interpretation of the data—or as expressed by Clarke et al.—achieving the minimal goal of semantic equivalence and the aspirational goal of conceptual equivalence (Clark et al., 2017).

## Conclusion

OACs have been the foci of this study but our results, in several ways, have implications for not only them but aging families in general. Being an unusual yet growing phenomenon, the dynamic of simultaneously aging family generations presents new challenges and demands for old-age care, particularly in terms of the arrangement of long-term care. There are three areas we would like to highlight. First, loneliness clearly stands out as a public health challenge and relates closely to the issue that care networks are dynamic and constantly evolving leading to precarious aging trajectories. In our study, OACs tended to extend their care networks to places and spaces outside their traditional comfort zones. Policies should be responsive to this. Second, moving to formality should not imply that informal networks should be replaced. Formally-arranged care initiatives should be more sensitive to working with informal family caretakers and understanding their needs, e.g., in terms of who are the most vulnerable to unmet care needs and understanding their “bargaining processes”. And third, as long as such an understanding does not feed into the formulation of policies, it remains unlikely that old-age care models and LTC systems can be sustainable and equitable. In general, old-age care policies do not exist in isolation but should be in tune with the policies guiding other multiple sectors such as the general health care, pension and retirement policies.

## Data Availability

The datasets presented in this article are not readily available because participants did not consent for their data to be shared publicly. Inquiries should be directed to julia.schroders@umu.se.

## Footnotes

1 The *podes* statistics provide information about Indonesia’s+/−65,000 villages. It is surveyed by Statistics Indonesia within periodic censuses (agriculture, economy, population) and covers the following areas: population, labor force, housing, settlements, environment, socio-culture, land use, economy, agriculture, trade and industries, education facilities, recreation, health facilities, nutrition, family planning, transportation, communication, security, and head of village information [Badan Pusat Statistik (BPS) Indonesia (Statistics Indonesia), 2016].
